# Functional proteomic characterization of cancer cell lines

**DOI:** 10.18632/oncoscience.351

**Published:** 2017-06-10

**Authors:** Wei Zhao, Jun Li, Gordon B. Mills

**Affiliations:** Department of Systems Biology, The University of Texas MD Anderson Cancer Center, Houston, TX 77030, USA

**Keywords:** cancer cell lines, proteomics, reverse-phase protein array, biomarker

Recent large-scale cancer genomic studies such as The Cancer Genome Atlas (TCGA) and International Cancer Genome Consortium (ICGC) have characterized the molecular portraits of tens of thousands of tumors, and provided unprecedented knowledge on the oncogenic lesions that may be used to guide cancer therapy. To capture the molecular features and identify links with drug response, cancer cell lines remain the most highly characterized and high-throughput model system in use. An inherent question is to what extent cell lines recapitulate molecular and clinical diversity of *in vivo* diseases.

Since the first large pharmacogenomics initiative of NCI-60 to analyze 60 cell lines [1], several studies such as the Cancer Cell Line Encyclopedia (CCLE) [2] and Catalogue of Somatic Mutations in Cancer (COSMIC) [3] have generated mutation, copy number and transcriptomic catalogues of over 1000 cell lines, and drug screening data of hundreds to thousands of compounds have been made available in public database such as Cancer Therapeutics Response Portal (CTRP) [4] and Genomics of Drug Sensitivity in Cancer (GDSC) [5]. A missing piece in the emerging molecular portrait of cancer cell lines is the protein expression profile. Proteins serve as the major functional unit in the cell and the therapeutic target of most drugs. Unfortunately protein expression levels, modifications and functions are poorly imputed by genomic or transcriptomic analysis. Our study [6] provides functional proteomics of over 650 independent cell lines representing the first comprehensive effort to address this need.

In the MD Anderson Cell Lines Project (MCLP), normalized protein expression data of 706 cell lines (including 651 independent cell lines encompassing 19 lineages) were generated using reverse-phase protein arrays (RPPA), the same platform employed to characterize over 8,000 tumors in TCGA (http://tcpaportal.org/tcpa/). Each cell line was profiled with an average of 230 high-quality antibodies that target total and phosphorylated proteins. The proteins are selected to provide a sparse unbiased coverage of all major functional and signaling pathways of relevance to cancer. A large subpopulation of these cell lines have been annotated at genomic and transcriptomic level and tested for drug sensitivity. A user-friendly web platform (http://tcpaportal.org/mclp/) was implemented to enable exploration of RPPA and related pharmacogenomics data. In addition to the rich information content, the most intriguing feature of MCLP is its integration with other large-scale multilayer data which will provide opportunities to advance our knowledge of protein features that define tumor subpopulations, protein network rewiring regulated by oncogenic lesions at DNA or RNA level, protein markers that predict therapeutic sensitivity, and identification of compounds likely to be active in select patient populations.

Investigation of protein expression pattern in MCLP and TCGA [7] both revealed lineage-dominant tumor/cell line classes and classes defined by molecular features that cross tumor lineage. Some heterogeneous tumor types were captured by distinct subsets of cell lines, such as the luminal, basal and claudin-low clusters of breast cell lines. The multi-lineage clusters support a pan-cancer approach to investigate specific pathways like Epithelial-to-Mesenchymal Transition (EMT) and squamous differentiation. With advanced methodologies to reduce tissue-specific signatures computationally, cell lines defined by molecular features rather than anatomic origins may provide useful models to explore cross disease hypotheses.

Integrated analysis of proteomic and transcriptomic data from cell lines revealed moderate quantitative correlation and concordance in co-expression networks, which reinforced the limitation of using transcriptional profiles as surrogates for protein function. The combined analysis of both information levels is better able to capture compensatory feedback loops and posttranslational regulation, thus allows more accurate modeling of signaling and pathway activity. Similarly, mutations in cancer driver genes, as indicators of altered pathways, are frequently used to dictate treatments. However, the rewired signaling pathways may elicit indirect, sometimes unexpected effects that contribute to ultimate resistance and/or provides combinatorial therapeutic opportunities. These mutation-protein associations are now readily investigated in this large collection of highly characterized cell lines. Our analysis revealed that cell lines demonstrate the functional consequences of frequently mutated pathways like PI3K and p53 signaling in concordance with tumors.

Identification of markers predictive of clinical benefit is a core goal for drug screening. A community effort [8] analyzing breast cell lines demonstrated that multi-omics profiling data provided non-redundant predictive information and that RPPA-based protein quantification and gene expression data showed similar high performance. Integration of MCLP with other pharmacogenomics datasets significantly expanded the collection of cell lines assayed for both gene and protein abundance, and therapeutic response. The complementary predictive power of protein and RNA levels is remarkable across all compound families. Of note, phosphorylated proteins are direct indicators of protein activity and provided unique predictive information in our study. Taken together, these datasets demonstrate the promise of improved –omic predictors to tailor cancer treatments.

In MCLP study, we explored therapeutic sensitivity of cell lines expressing protein markers of EMT pathways. Surprisingly a strong EMT signature was observed in cell lines from nearly all lineages assessed in MCLP. As expected, EMT was associated with resistance to the majority of therapeutic modalities. However, cells with a strong EMT signature demonstrated marked sensitivity to HMGCR targeting compounds and moderate sensitivity to a subset of drugs targeting the RAS/MAPK suggesting potential treatment options for this hard to treat population.

The RPPA data and the interactive web portal collectively represent opportunities to explore tumor protein features in cancer cell lines and to capitalize on existing pharmacogenomics data to facilitate drug implementation.
